# Intracellular Delivery of siRNA by Polycationic Superparamagnetic Nanoparticles

**DOI:** 10.1155/2012/218940

**Published:** 2012-08-30

**Authors:** Betzaida Castillo, Lev Bromberg, Xaira López, Valerie Badillo, Jose A. González Feliciano, Carlos I. González, T. Alan Hatton, Gabriel Barletta

**Affiliations:** ^1^Department of Chemistry, University of Puerto Rico at Humacao, Humacao 00791, Puerto Rico; ^2^Department of Chemical Engineering, Massachusetts Institute of Technology, Cambridge University, MA 02139, USA; ^3^Department of Biology, University of Puerto Rico at Río Piedras, San Juan 00931, Puerto Rico

## Abstract

The siRNA transfection efficiency of nanoparticles (NPs), composed of a superparamagnetic iron oxide core modified with polycationic polymers (poly(hexamethylene biguanide) or branched polyethyleneimine), were studied in CHO-K1 and HeLa cell lines. Both NPs demonstrated to be good siRNA transfection vehicles, but unmodified branched polyethyleneimine (25 kD) was superior on both cell lines. However, application of an external magnetic field during transfection (magnetofection) increased the efficiency of the superparamagnetic NPs. Furthermore, our results reveal that these NPs are less toxic towards CHO-K1 cell lines than the unmodified polycationic-branched polyethyleneimine (PEI). In general, the external magnetic field did not alter the cell's viability nor it disrupted the cell membranes, except for the poly(hexamethylene biguanide)-modified NP, where it was observed that in CHO-K1 cells application of the external magnetic field promoted membrane damage. This paper presents new polycationic superparamagnetic NPs as promising transfection vehicles for siRNA and demonstrates the advantages of magnetofection.

## 1. Introduction

Small interfering RNA's (siRNAs) are short double-stranded nucleic acids, commonly containing 19–21 residues and 3′-dinucleotide overhangs, which are widely used as synthetic reagents to reduce gene expression of target RNA in cells [1] and hence prevent the synthesis of specific proteins [2]. siRNAs are being developed to target therapeutically important genes involved in cancer, viral infections, autoimmune and neurodegenerative diseases [3]. However, these short double-stranded nucleic acids are unstable within the extracellular environment, they cannot cross cell membranes and due to their small size are readily secreted by the renal system [2, 4]. Progress to overcome some of these obstacles has been made using viral and synthetic vectors [5–10]. However, there is no universally accepted method for siRNA delivery, since all vectors exhibit limitations [11]. A good carrier must meet several requirements: (a) facile formation of a complex with siRNA, (b) crossing of the cell membrane, (c) the complex must be released in the cytoplasm from endosomes and release its siRNA cargo, and (d) the carrier has to be nontoxic [11]. Since siRNAs have large negative charge densities, polycationic carriers such as poly(ethylene imine) (PEI) have been shown to be good transfection vehicles, however, high-charge densities seem to make this type of materials toxic to most cell lines [12]. An additional quality, especially for *in vivo* delivery, is that the material should target the desired tissue, and for this, magnetofection has shown potential [13]. Several studies have demonstrated that magnetofection can efficiently deliver siRNA to living cells cultivated *in vitro *[14–16], and it appears to be a reliable and gentle method for siRNA and DNA delivery into difficult to transfect cells such as mammalian fibroblasts [17]. For magnetofection, polycationic paramagnetic nanoparticles (NPs) are coupled to a nucleic acid through electrostatic interactions to form magnetic vectors that can be rapidly drawn to, and concentrated on the surface of the target cells using the attractive force of an externally applied magnetic field. This facilitates the uptake of the magnetic vector into the cell endosomes [18, 19]. Some of the advantages of this technique over nonmagnetic approaches are (i) improved transfection efficiency via lowering the diffusion barriers [19–22] and (ii) the possibility of site-specific delivery by focused application of a magnetic field gradient [23, 24]. Recent studies have demonstrated significant enhancement of siRNA uptake through the application of magnetofection [25]. *In vivo* magnetic-field-guided local transfection in the gastrointestinal tract and in blood vessels has also been demonstrated [24]. From the magnetic material viewpoint, magnetite (Fe_3_O_4_) surface-modified by biocompatible polymers can be utilized in magnetofection, because of its relatively low toxicity [26–28], high saturation magnetization (up to 92 emu/g [29]), and well-developed methods of synthesis [30, 31]. Several reports on toxicity of iron oxide NP used in magnetofection have been published [17]. Evaluation of the cytotoxicity of hexanoyl chloride-modified, chitosan-stabilized iron oxide NP showed that even at NP concentrations 50-fold higher than the concentration required for high efficiency of transfection, NPs display no negative effect on the cell viability [32]. Superparamagnetic iron oxide NP appear to be biodegradable when injected intravenously, and the iron from the NP is introduced into the normal plasma iron pool and can be incorporated into hemoglobin in erythrocytes or used for other metabolic processes [33]. Upon internalization of the magnetic NP into cells, with time, iron can be released into the intracellular compartment and participate in the cellular iron metabolism [34, 35]. Application of an external magnetic field for the targeted delivery of siRNA complexes with magnetic NP to a tumor, could selectively downregulate the expression of a gene of choice in these cells without affecting healthy ones, making this approach an attractive cancer therapeutic strategy by reducing side effects while lowering the cost of therapy [17]. However, this method is still in its initial stages of development and new magnetic nanoparticles to lead optimal siRNA delivery, including improved intracellular targeting while reducing cytotoxic effects are needed [36]. 

As previously mentioned, cationic poly(ethylene imine) (PEI) is an efficient delivery system of siRNA in a variety cell lines and *in vivo* [7, 37–44]. Evaluation of several linear and branched PEI structures with molecular weights ranging from 0.8 to 25 kDa, for siRNA delivery, showed that 25 kDa branched PEI was the most efficient transfection vehicle [25, 33]. However, the high transfection efficiency of the large, branched PEI species is associated with high cytotoxicity [45–49] due to necrosis [47]. In the present work, we aimed at evaluating the magnetofection properties of two types of polycationic core-shell nanoparticles (~200 nm), wherein the magnetite core and functional siloxane shell covalently linked to PHMBG or PEI. Synthesis, magnetic properties, and bactericidal action of such NPs have been previously reported [29, 50–52]. The NPs transfection efficiency was evaluated by conducting a knockdown efficiency study using a dual luciferase reporter assay, and toxicity was studied by the cell proliferation (MTS) and by the lactate dehydrogenase assay (LDH) in HeLa and CHO-K1 cells lines. We found that PEI's toxicity was reduced and its transfection efficiency (in one cell line) improved when attached to the surface of the superparamagnetic nanoparticles. Magnetofection increased the efficiency of these transfection vehicles even further.

## 2. Materials and Methods

### 2.1. Materials

Branched polyethyleneimine (PEI, nominal average molecular weight, 25 kDa) with a molar ratio of primary to secondary to tertiary amino groups of 1 : 2 : 1 was obtained from Sigma-Aldrich Chemical Co. (St. Louis, MO). After dialysis in water (MWCO 12–14 kDa) and removal of lower molecular weight fractions, the *M*_*w*_ was 38 kDa and *M*_*w*_/*M*_*n*_ = 1.55. Poly(hexamethylene biguanide) (PHMBG) was from Arch UK Biocides Ltd. (Manchester, UK) supplied as a 20 wt% aqueous solution (Cosmocil CQ) with a reported *M*_*w*_ of 2674 Da and a polydispersity of 1.89. FeCl_3_·6H_2_O (98%), FeCl_2_·4H_2_O (99%), aqueous 25 wt% glutaraldehyde, tetraethyl orthosilicate (99%, TEOS), 3-glycidoxypropyl trimethoxysilane (97%, GPTMS), and Triton X-100 solution, were all purchased from Sigma-Aldrich Chemical Co. (St.Louis, MO). Fetal bovine serum (FBS) was obtained from Thermo Scientific Hyclone (Logan, Ut). Ethidium bromide was purchased from Bio-Rad (Hercules, CA). CHO-K1 and HeLa cell lines were obtained from ATCC (Manassas, VA). Dual reporter luciferase assay and cell titer 96 aqueous solution were purchased from Promega (Madison, Wisconsin). Cell culture media, penicillin, streptomycin, optiMEM buffer, phosphate buffered saline (PBS), trypsin and trypan blue, were all purchased from Invitrogen (Frederick, MD). The Firefly siRNA sequence and negative control siRNA are commercially available from Applied Biosystems (catalog#AM4629). LDH Cytotoxicity Detection Kit was purchased from Clontech (Mountain View, CA). Magnets (NeFeB, magnetic strength of 0.3 Tesla) were purchased from Chemicell, “MagnetoFACTOR-96 plate.”

### 2.2. Magnetic Particle Synthesis

*Magnetite-silica core-shell particles functionalized with epoxy groups *were synthesized as described in our previous work [29]. First, magnetite particles were prepared, which were well-dispersed in water with the aid of tetramethylammonium hydroxide (TMAOH). In the second step, the magnetic particles were encapsulated by a functional shell comprising tetraethyl orthosilicate (TEOS) and epoxy-functional 3-glycidoxypropyltrimethoxysilane (GPTMS). The third step comprised attachment of either PEI or PHMBG chains. Thus, FeCl_3_ *·*6H_2_0 (7.58 g, 28 mmol) and FeCl_2_ *·*4H_2_O (2.78 g, 14 mmol) were dissolved in 25 mL DI water and the solution was brought to 80°C under nitrogen purge within ~30 min. The solution was poured into 25 mL of 30% NH_4_OH and the ensued black precipitate was stirred and kept at 80°C for 1 h. The resulting particle suspension was sonicated for 1 min and separated from supernatant by magnetocollection. The particles were then placed into a tube containing 30 mL of 0.33 M aqueous solution of TMAOH. The suspension was observed to be stable. The suspension was separated by magnetocollection and washed twice with 50 mL of deionized water. The resulting TMAOH-stabilized magnetite suspension (~25 mL) was diluted with (in) 40 mL ethanol. To the resulting suspension, 3.6 mL (16 mmol) of TEOS were added and the suspension was sonicated for 5 min, followed by addition of 4.6 mL (20 mmol) of GPTMS. The suspension was kept under vigorous shaking at room temperature for 48 hours and the particles were separated using magnetocollection, dialyzed (MWCO 12–14 kDa) against an excess of deionized water overnight, snap frozen, and lyophilized. The resulting epoxy-modified particles designated M/SiO_2_ were characterized by FTIR and TGA. Elemental analysis were found (%) : C, 17.0; Fe, 24.1; N, 0.04. The M/SiO_2_ particles were stored at −20°C prior to use.

*Core-Shell Particle Modified with PHMBG *(PHMBG-M/SiO_2_). Particles designated PHMBG-M/SiO_2_ were synthesized using 10 mL of the aqueous M/SiO_2_ particle suspension (magnetite content, ~0.5 g), to which a solution of 0.9 g of PHMBG in 500 mL of deionized water was added. The mixture was sonicated for 5 min and kept at 80°C for 16 h followed by shaking at 250 rpm at room temperature for 2 days, followed by dialysis against deionized water (MWCO, 12–14 kDa) and drying by lyophilization. The resulting particles were characterized by elemental analysis, TEM, DLS, SQUID, and TGA. Elemental analysis, found (%) : C, 27.8; H, 5.83; Fe, 18.3; N, 19.7. 

*Core-shell particles modified with PEI *(PEI*- *M/SiO_2_) were synthesized as follows. To the TMAOH-stabilized magnetite suspension (~25 mL) prepared as described above, 40 mL of absolute ethanol were added and the diluted suspension was sonicated for 1 min. To the resulting suspension, 3.6 mL (16 mmol) of TEOS were added and the suspension was sonicated for 5 min, followed by addition of 4.6 mL (20 mmol) of GPTMS. The suspension was shaken (200 rpm) at room temperature for 1 h, aqueous solution of PEI (5 g in 100 mL water) was added, and the resulting mixture was shaken at room temperature for 1 h, kept at 80°C for 1 h and then shaken at room temperature for 16 h. The suspension was then dialyzed (membrane MWCO, 12–14 kDa) against excess deionized water. The resulting suspension did not exhibit any visible sedimentation of particles for several days at rest. The resulting PEI-M/SiO_2_ particles were separated by magnetocollection, snap frozen, and lyophilized. Elemental analysis, found (%) : C, 43.5; Fe, 7.92; N, 21.1.  Table 1 lists some of the properties of these materials.

### 2.3. Relative Binding Affinity Assay

#### 2.3.1. Ethidium Bromide Displacement Assay

Ethidium bromide (EtBr, 1 *μ*g) was added to 100 *μ*L of MEM medium in the fluorescence cell. Fluorescence was recorded at an excitation wavelength of 485 nm and an emission wavelength range of 590 nm. siRNA (2.2 *μ*g) was added, and the fluorescence remeasured. An aliquot of polymer was then titrated into the solution to a certain N/P ratio. Samples were gently mixed, and readings were taken after 15 min of incubation. The relative fluorescence (RelFlu) was calculated as follows (fluorescence = fluo., and NP = polymer nanoparticle):

(1) $$\begin{array}{c} \text{RelFlu}\;=\;\frac{\left[\text{fluo}.\;\left(\text{EtBr}+\text{siRNA}+\text{NP}\right)-\text{fluo}.\;\left(\text{EtBr}\right)\right]}{\left[\text{fluo}.\;\left(\text{EtBr}+\text{siRNA}\right)-\text{fluo}.\;\left(\text{EtBr}\right)\right]}. \end{array}$$

The fluorescence intensity of EtBr increases as it intercalates with the bases (of siRNA) forming strong complexes. Polymers interacting with siRNA displace EtBr and, therefore, the observed relative fluorescence decreases—this is indicative of a polymer that forms a strong complex with siRNA.

### 2.4. Transfection Efficiency

#### 2.4.1. Cell Culture Assays

Experiments were carried out using CHO-K1 and HeLa cells. CHO-K1 cells were grown in F-12K medium with L-glutamine containing 10% fetal bovine serum (FBS) and 1% penicillin. HeLa cells were cultured in MEM medium with L-glutamine supplemented with 10% FBS and 1% penicillin. Both cells were incubated at 37°C and 5% CO_2_.

#### 2.4.2. Luciferase Reporter Plasmids

The Firefly Luciferase mammalian expression vector was constructed by cutting pSP-Luc+ vector (Promega) with *Kpn1*/*Xba1*, and cloning the Luc sequence into pCDNA 3.1+ (Invitrogen). The pRL-CMV vector containing the *Renilla *luciferase reporter was purchased from Promega and used as internal transfection control. 

#### 2.4.3. Particle-siRNA and Particle-DNA Complexes Formation and Cell Transfection

PEI, PEI-M/SiO_2_, PHMBG, and PHMBG-M/SiO_2_ stock solutions or suspensions (0.9 mg/mL) were prepared in PBS (pH 7.2). N/P ratios were calculated considering all amino groups on PEI and PEI-M/SiO_2_, and all biguanide groups on PHMBG and PHMBG-M/SiO_2_. 

**Figure 1:**
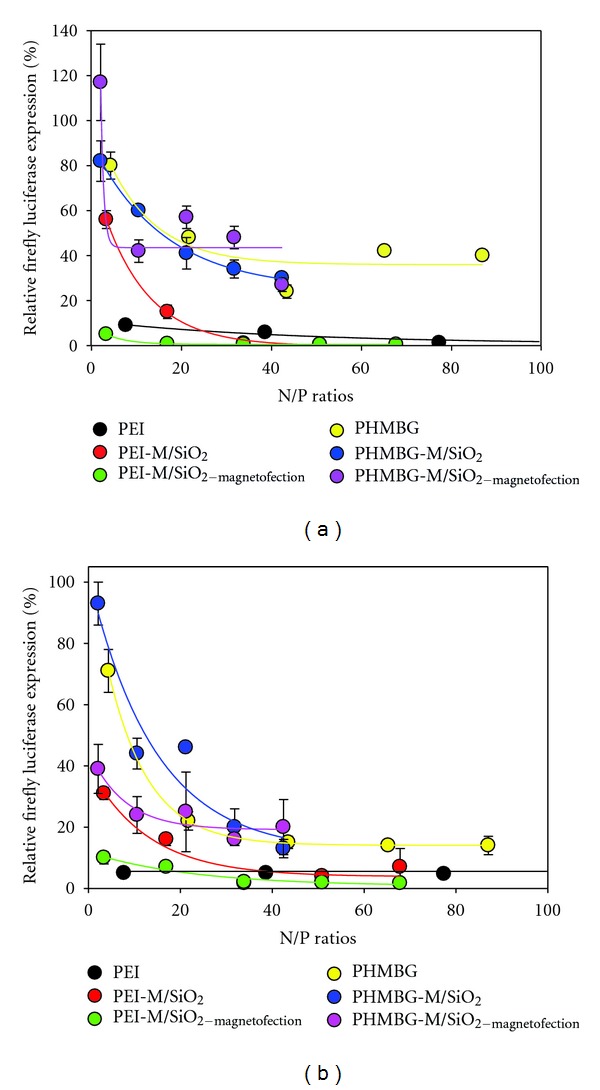
Effect of nanoparticle/siRNA (N/P) ratio on the transfection efficiency of all materials in CHO-K1 (a) and HeLa (b) cell lines. Values represent mean ± standard error of the mean (SEM) from three independent transfections. Triplicates were normalized using *Renilla* luciferase as an internal transfection control.

For anti-Firefly siRNA and Firefly/Renilla plasmids DNA transfection using PEI, cells were grown in 12-well plates at an initial density of 14 × 10^4^ to 17 × 10^4^ cells per well in 1 mL of penicillin free F12K (CHO-K1) or MEM (HeLa) medium supplemented with 10% FBS to be 60–70% confluent at the time of transfection. After 24 h of plating, 50 *μ*L of a solution containing the PEI-siRNA and PEI-DNA complexes were added to each well. This solution was prepared as following: the appropriate amount of PEI was mixed with 70 pmol of firefly siRNA, 6.0 *μ*g of Firely luciferase DNA, 1.0 *μ*g of Renilla luciferase DNA, and resuspended in OptiMEM I buffer. The mixture was kept at room temperature for 1 h prior to transfection. After 24 h of transfection, the cells were lysed with passive lysis buffer and analyzed for Firefly luciferase and Renilla luciferase expression using a Dual luciferase Reporter assay kit (Promega, Madison, WI). The Firefly luciferase/Renilla luciferase luminescence intensity ratio (FRR) was calculated. To quantify gene knockdown, the FRR from cells transfected with siRNA polyplexes containing anti-Firefly luciferase (GL2 + GL3) siRNA were compared with identical polyplexes containing a negative control siRNA. All values shown on Figure 1 are relative to the firefly luciferase expression of cells transfected with a negative control siRNA sequence. Relative firefly luciferase expression (%) = FRR of cells transfected with siRNA polyplexes containing anti-Firefly/FRR of cells transfected with negative control siRNA polyplexes.

For anti-Firefly siRNA transfection using PEI-M/SiO_2_, PHMBG and PHMBG-M/SiO_2_ as carriers, the Firefly/Renilla plasmids DNA were first transfected using PEI. The cells were grown as previously described. At the same time of plating, the PEI-DNA complex was added to each well. PEI-Firefly/Renilla plasmids DNA complexes were prepared as follows: 10 *μ*L of PEI stock solution (0.9 mg/mL) was mixed with 6.0 *μ*g of Firely luciferase DNA, 1.0 *μ*g of Renilla luciferase DNA and resuspended in OptiMEM I buffer. The mixture was kept at room temperature for 1 h prior to transfection. After 24 h of transfection, the culture media were removed and the cells were washed with PBS. Then, fresh media and polymer/anti-Firefly siRNA complex were added to each well. The complexes of PEI-M/SiO_2_, PHMBG and PHMBG-M/SiO_2_, with anti-Firefly siRNA were formed by mixing the appropriate amount of polymer stock solution (0.9 mg/mL) with 70 pmol of firefly siRNA and OptiMEM buffer. The mixture was kept at room temperature for 30 mins prior to transfection. After 24 h of transfection, cell lysates were formed and analyzed for luciferase activity as previously described. 

*In vitro* magnetofection was carried out applying a magnetic field under the cell-culture plate to concentrate particles into the target cells, using the same procedure as described above with only minor modifications: cells were exposed to a magnetic field using the MagnetoFACTOR-96 plates (Chemicell GmbH, Berlin, Germany; magnetic field, 0.3 Tesla).

### 2.5. Cell Proliferation Assay

#### 2.5.1. MTS

For cell viability, the 3-(4,5-dimethylthiazol-2-yl)-5-(3-carboxymethoxyphenyl)-2-(4-sulfophenyl)-2H-tetrazolium (MTS) assay was employed. Cells (40000 cells/well) were seeded into 96-well microtiter plates (100 *μ*L of penicillin free culture medium with 10% FBS). After 24 h, culture media were replaced with culture media containing serial dilutions of polymer solutions, and the cells were incubated for 24 h. 20 *μ*L of MTS was subsequently added to each well. After 2 h, the optical intensity of each was measured spectrophotometrically at a wavelength of 490 nm in a microplate reader. The spectrophotometer baseline was calibrated using culture medium without cells. For PEI-M/SiO2 and PHMBG-M/SiO2, the assay was performed with and without the external magnetic field (magnetofection) provided by the magnetic plates. Hereafter, transfection of PEI-M/SiO2 and PHMBG-M/SiO2 by magnetofection will be referred as to PEI-M/SiO_2-magnetofection_ and PHMBG-M/SiO_2-magnetofection_.

The relative cell viability was calculated with untreated cells as a control using the following equation:

(2) $$\begin{array}{c} \text{relative}\;\text{cell}\;\text{viability}\;\left(\%\right)\;=\;\left\{\frac{\left[\left(\text{abs}\right)\text{treated}\right]}{\left[\left(\text{abs}\right)\text{untreated}\right]}\right\}\;\times \;100. \end{array}$$

### 2.6. Cytotoxicity

#### 2.6.1. LDH

The plasma membrane damage has been assayed by quantifying the release of lactate dehydrogenase (LDH), a stable cytoplasmic enzyme normally not secreted outside of the cells. For detection of LDH, the Cytotoxicity Detection Kit (Clontech, Mountain View, CA) was used. Cells (40000 cells/well) were seeded into 96-well microtiter plates (100 *μ*L of penicillin free culture medium with 1% FBS). After 24 h, culture media was replaced with fresh one before addition of the polymers. The polymer dilutions were added to the appropriate weal and cells were incubated for 24 h. The 96-well plate was centrifuged and 100 *μ*L of the supernatant was transferred to the corresponding wells of an optically clear 96-well flat-bottom plate. 100 *μ*L of the reaction mixture, containing the tetrazolium salt, was then added to each well and incubated for 30 minutes at room temperature. The LDH concentration in the cell culture supernatant was determined spectrophotometrically at a wavelength of 492 nm in a microplate reader (Thermo Electron Corp., Vantaa, Finland). For PEI-M/SiO_2_ and PHMBG-M/SiO_2_, the assay was performed with and without the external magnetic field. Cytotoxicity (%) was calculated using the level of spontaneous LDH release from untreated cells as a low control and the maximum of LDH activity that can be released from the 100% dead cells (in response to Triton X-100) as a high control:

(3) cytotoxicity  (%)  = {[(abs)sample  −  (abs)low  control][(abs)high  control  −  (abs)low  control]}  ×  100.

## 3. Results and Discussion

**Scheme 1:**
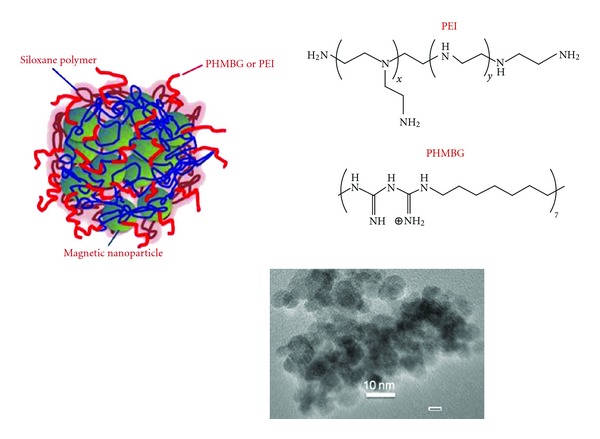
Structure of the core-shell superparamagnetic nanoparticles utilized in the present study.

Scheme 1 depicts a cartoon illustrating the structure of the NPs employed in this study. Based on elemental analysis, TGA results and structure modeling, the content of biguanide groups in the PHMBG-M/SiO_2_ particles was estimated to be approximately 2.3 mmol/g, while the amino groups content of the PEI-M/SiO_2_ particles was ca. 3.2 mmol/g [29]. These values were used to estimate the ratio of the positively charged groups of the particles to the number of phosphate groups on the siRNA (N/P ratios). Transfecting properties of the vectors for Silencer Firefly luciferase (GL2 + GL3) siRNA were studied in HeLa and CHO-K1 cell lines. The materials used were PEI-M/SiO_2_, and PMBG-M/SiO_2_ particles and PHMBG solutions, whereas PEI solutions were used as controls. 

First, we investigated the delivery efficiency of these materials by conducting a knockdown efficiency study using a dual luciferase reporter assay. The luminescence intensities of the cell lysates were used to measure the siRNA cellular delivery efficiencies at different N/P ratios. For this, the gene for firefly luciferase and Renilla luciferase is transfected along with a negative control (nontargeting sequence) and a siRNA against firefly luciferase (targeting sequence).Therefore, the luminescence intensities or firefly luciferase expression of the control cells is expected to be higher than the experimental cells. High firefly luciferase expression means low gene knockdown efficiency. For this assay it is necessary to cotransfect the plasmid DNA encoding firefly and Renilla luciferase. Our initial results demonstrated that PEI-M/SiO_2_, PHMBG, and PHMBG-M/SiO_2_ materials were not able to transfect DNA (data not shown), and, therefore, a double transfection assay was employed using PEI as the transfecting vehicle for both firefly and Renilla luciferase DNA. As shown in Figures 1(a) and 1(b), the lysate of CHO-K1 and HeLa cells treated with PEI-M/SiO_2_ without the presence of an external magnetic field (no magnetofection) showed a dose-dependent trend. Specifically, increasing the N/P ratios decreased the firefly luciferase expression, and PEI-M/SiO_2_ particles were a less efficient transfecting vehicles than PEI for both cell lines, at low N/P ratios. However, at low N/P ratios, magnetofection (which helps to concentrate the NPs on the cell surface [53]) improved the transfection efficiency of PEI-M/SiO_2_-siRNA complex in CHO-K1 cells over PEI (PEI_N/P: 8_ = 91%; PEI-M/SiO_2  N/P: 3_ = 45%; PEI-M/SiO_2-magnetofection  N/P: 3_ = 95%—Figure 1(a)). At N/P ratios higher than 34, magnetofection did not improve the transfection efficiency any further than 99% (Figure 1(a)). Although for HeLa cells magnetofection also improved the transfection efficiency of PEI-M/SiO_2_ at low N/P ratios, the transfection efficiency was still less than that observed with PEI, but when N/P ratios higher than 34 were used, magnetofection did improve the transfection efficiency of PEI-M/SiO_2_ over PEI slightly (PEI_N/P: 155_ = 94%; PEI-M/SiO_2  N/P: 68_ = 93%; PEI-M/SiO_2-magnetofection  N/P: 68_ = 98%, Figure 1(b)).

PHMBG, PHMBG-M/SiO_2_, and PHMBG-M/SiO_2-magnetofection_  were less efficient transfecting vehicles of siRNA compared to the control PEI (Figure 1). The silencing effect was manifested as a dose-dependent decrease in firefly luminescence, with up to 60%, 70%, and 73% downregulation of firefly luciferase expression in CHO-K1 cells and up to 86%, 87%, and 50% in HeLa cells by using PHMBG, PHMBG-M/SiO_2_, and PHMBG-M/SiO_2-magnetofection_, respectively.

**Figure 2:**
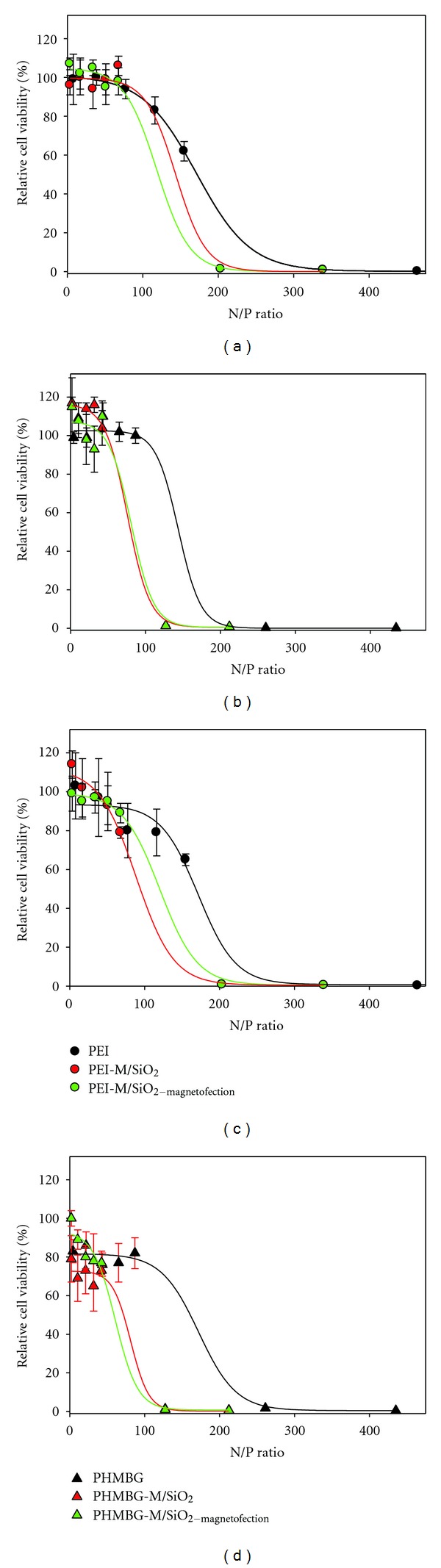
Effect of nanoparticle/siRNA (N/P) ratio on metabolic activity in CHO-K1 ((a) and (b)) and HeLa ((c) and (d)) cell lines, as a function of polymer/siRNA (N/P) ratios. The cell viability was determined by MTS assay and was shown as the mean. Error bars are the standard deviation of eight determinations. Relative cell viability was calculated using untreated cells as a control.

The effect of these particle/siRNA complexes on CHO-K1 and HeLa cell lines' metabolic activity was measured using the MTS assay, measuring the buildup of the formazan product at 490 nm, which is directly proportional to the number of living cells in the culture. Although previous studies have demonstrated that PEI induces cytotoxicity [54, 55], our results (shown in Figure 2) revealed that in the range of concentrations used for siRNA transfection, PEI, and the rest of the tested materials did not promote cell death (at N/P ratios up to 60 viability of the cells was close to that of untreated ones) in both CHO-K1 and HeLa cells lines. However, above an N/P ratio of 200 all materials tested caused cell death (Figure 2). At an N/P = 200, the toxicity of all materials are indistinguishable from that of PEI.

**Figure 3:**
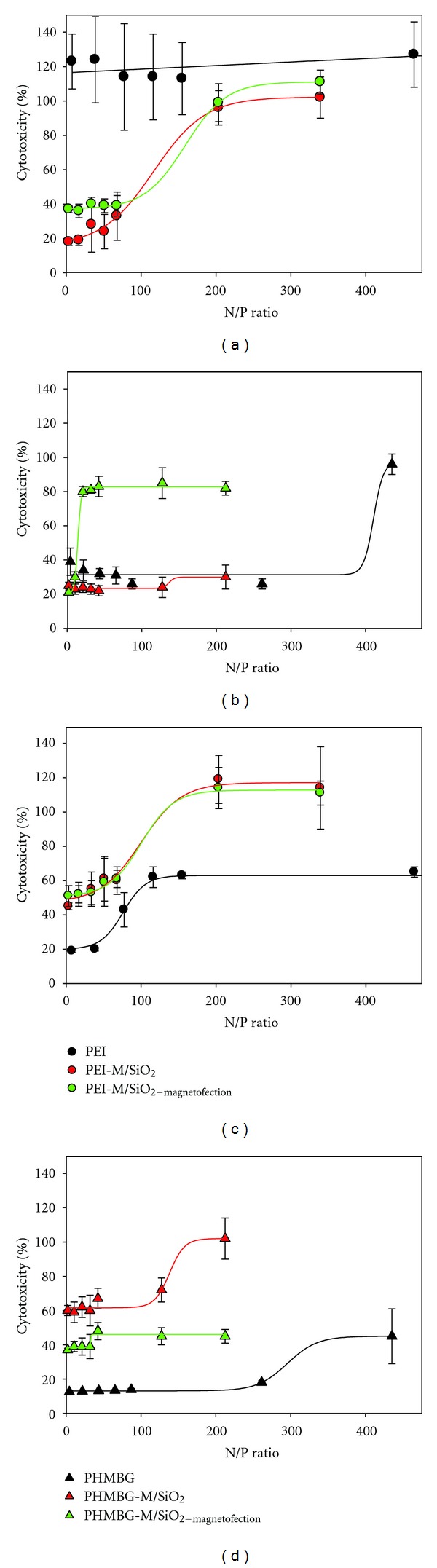
Effect of nanoparticle/siRNA (N/P) ratio on membrane permeability of CHO-K1 ((a) and (b)) and HeLa ((c) and (d)) cell lines.

These results suggest that the dose-dependent and the observed differences in siRNA transfection efficiency among the nanoparticle vehicles (highlighted in Figure 1), are unrelated to cell viability. Furthermore, contrary to previous studies, siRNA was not toxic at the concentrations used in this study [56]. Next, we investigated the effects of the particles and polymers under study on the cell membrane integrity (cytotoxicity) using the LDH assay (see Section 2). These experiments were carried out under similar conditions as the MTS assay, where CHO-K1 and HeLa cells were exposed to various N/P ratios of the NPs complexes. As shown in Figures 3(a) and 3(b), up to the N/P ratios of about 40 wherein optimum siRNA transfection was observed, PEI induced the most membrane damage to CHO-K1 cells. The remainder of the NPs possessed cytotoxicity ranging from 20 to 40%. Notably, PHMBG-M/SiO_2-magnetofection_ versus PHMBG-M/SiO_2_ showed an increase in cytotoxicity from 30 to 80% when the N/P ratio was increased from 10 to 20 due to the influence of the external magnetic field (Figure 3(b)). However, the external magnetic field did not significantly affect the cytotoxicity of PEI-M/SiO_2_. These results suggest that PEI's siRNA transfection efficiency (Figure 1(a)) could be due to disruption of the membrane (cytotoxicity). As shown in Figure 3(a), attaching cytotoxic PEI to the magnetic NPs reduced its cytotoxicity. At the highest N/P ratios employed, PEI and PEI-M/SiO_2_ with or without the external magnetic field significantly enhanced the membrane damage in CHO-K1cells, showing dose-dependent LDH release (Figure 3(a)). No NP dose dependence was observed on membrane permeability of CHO-K1 cells with PHMBG and PHMBG-M/SiO_2_ (except for PHMBG-M/SiO_2-magnetofection_, as previously mentioned—Figure 3(b)). In contrast, for HeLa cells all materials used in the study (with and without an external magnetic field) showed dose-dependent LDH release (Figures 3(c) and 3(d)). Surprisingly, the amount of LDH released in the presence of PEI-M/SiO_2_ with and without an external magnetic field was higher than with PEI at all of the N/P ratios employed. These results suggest that the membranes of HeLa cells were more resistant to PEI. Interestingly, the PHMBG-M/SiO_2_ particles were more cytotoxic than PHMBG in HeLa cells (Figure 3(d)). Although the external magnetic field helped reduce its cytotoxicity, the particles still remained more cytotoxic than PHMBG (Figure 3(d)).

**Figure 4:**
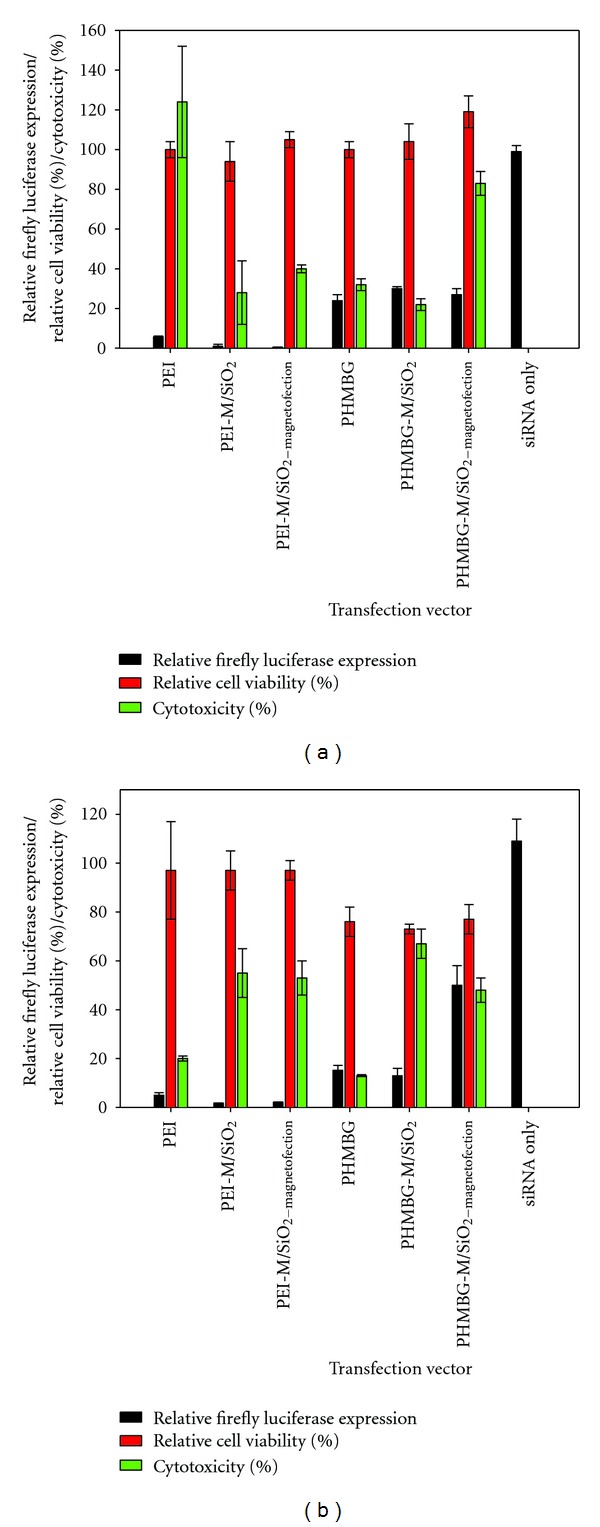
Panel (a) CHO-K1; (b) HeLa. N/P ratios: 39 for PEI, 34 for PEI-M/SiO_2_, 43 for PHMBG, and 42 for PHMBG-M/SiO_2_.

Comparing the transfection efficiency, cell viability, and cell membrane integrity of all materials at the optimum N/P ratios (from 34 to 43) for siRNA transfection shows that, although PEI is an efficient transfecting vehicle for CHO-K1 cells, it is highly cytotoxic (100% LDH released). Our results show that the PEI-modified PEI-M/SiO_2_ particles possessed higher transfecting potential and substantially reduced cytotoxicity than PEI. Application of the external magnetic field (PEI-M/SiO_2-magnetofection_) did not alter the cell viability or cytotoxicity of the particles, but it did significantly increase the transfection efficiency of PEI-M/SiO_2_ in CHO-K1 cells (Figure 4(a)). The siRNA transfection efficiency of PEI and PEI-M/SiO_2_ in HeLa cells was similar, and PEI-M/SiO_2-magnetofection_ did not improve the siRNA uptake at this particular N/P ratio. No decrease in cell viability and or increase in cytotoxicity were observed with PEI-M/SiO_2_ and PEI-M/SiO_2-magnetofection_ in HeLa cells (Figure 4(b)). Both in CHO-K1 and HeLa cells, PHMBG's NPs were less efficient transfecting vehicles than PEI's modified NPs, but in CHO-K1, they were less cytotoxic than PEI, whereas in HeLa they were more cytotoxic. Surprisingly, PHMBG-M/SiO_2-magnetofection_ caused significant membrane disruption to CHO-K1 cells (Figure 4(a)). Surprisingly, in HeLa cells, PHMBG-M/SiO_2-magnetofection_ was a less efficient transfecting vehicle than PHMBG-M/SiO_2_ (Figure 4(b)).

The last column in Figures 4(a) and 4(b) demonstrates that siRNA cannot cross cell membranes by itself, as demonstrated by the transfection of siRNA without any of the NP materials. 

**Figure 5:**
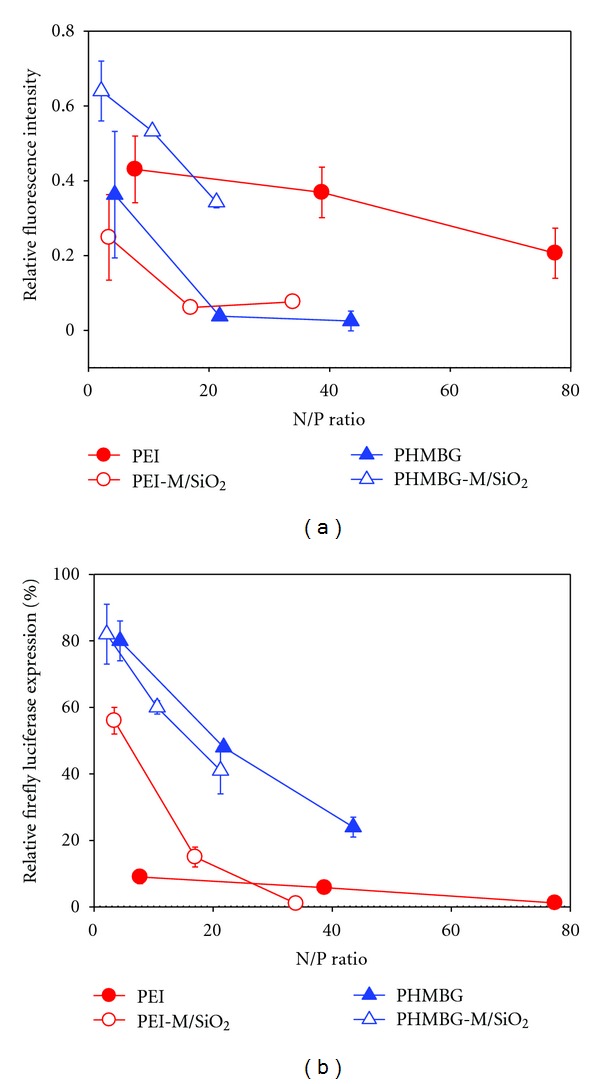
Effect of polymer: siRNA N/P ratios on the (a) relative binding affinity, and (b) the transfection efficiency. A decrease in fluorescence intensity (on a) correlates to increased binding between polymer/siRNA complexes. Note: the relative binding affinity was measured at the 3 lowest N/P ratios for all transfectin vehicles.

As previously discussed, an efficient delivery vehicle carrying siRNA across a cell membrane to downregulate the expression of the target gene requires the successful completion of several key steps [57, 58], the first one being the ability of the NPs to bind siRNA. Towards this end, we employed the ethidium bromide displacement assay to assess the relative degree of binding between the respective polyelectrolyte and siRNA. Our results show that increasing the N/P ratios also increase the binding between the delivery vehicle and siRNA (the relative fluorescence intensity decreases, Figure 5(a)). Concurrently, increasing the N/P ratio results in higher transfection efficiency on both cell lines (Figure 5(b) shows the results obtained with CHO-K1 cells at the same N/P ratios), which suggest a relationship between the degree of binding and transfection efficiency. Both transfection efficiency and degree of binding increase linearly for all materials used in the study. However, this does not mean that formation of a strong complex with siRNA will improve a particular vehicle's transfection efficiency. For example, although PEI-M/SiO_2_ forms a stronger complex with siRNA than PEI, the former is a less efficient transfecting vehicle at the lowest N/P ratios analyzed (Figures 5(a) and 5(b)).

Furthermore, PHMBG's show a slightly different trend than PEI's, in which the magnetite-modified- polycation (PHMBG-M/SiO_2_) is less effective than PHMBG in sequestering siRNA, but their transfecting efficiencies are similar. PEI's different complexation properties could perhaps be attributed to the particles' size differences: PEI-M/SiO_2_ is a much larger particle than PEI and forms clusters of about 200 nm, possibly increasing its siRNA complexation capacity. In terms of the differences in transfection efficiency between PEI-M/SiO_2_ and PEI, size and charge distribution differences between the two might benefit the latter. In the case of PHMBG's, biguanide groups are known bidentate chelators, and it is conceivable that PHMBG binds siRNA chelating the backbone phosphates. It is possible that this chelating ability is diminished in PHMBG-M/SiO_2_, since some of its biguanide groups are occupied by the SiO_2_ groups, yielding a weaker complexation capacity to siRNA. However, the above discussion is based on the EtBr assay results. Additional experiments are needed to test these hypotheses. In future studies, the complexation properties and transfection efficiency of these materials will be analyzed by confocal and transmittance electron microscopy. Regarding the effect of the transfecting vehicle on the cell membrane (cytotoxicity), our results show that on CHO-K1 cells, PEI-M/SiO_2_ causes significantly less membrane damage than PEI (Figure 3(a)).

Previous studies have demonstrated that electrostatic interactions are the main driving force for the formation of cationic components-type complexes with cell membranes [59–61]. We could assume that PEI possess higher positive charge density than PEI-M/SiO_2_ (since some of its sites are modified by SiO_2_ groups) which might induce excessive harmful electrostatic interactions with the membrane of CHO-K1 cells, as shown in Figure 3(a) at low N/P ratios. These excessive electrostatic interactions might disrupt the membrane enhancing PEI's transfecting ability. However, this idea cannot be substantiated in the current study, especially since two different cell lines were used. For example, Figures 3(c) and 3(d) reveal that PEI was not cytotoxic (at low N/P ratios) towards HeLa cells, and yet it showed to be a good transfection vehicle. Similar variance in cytotoxicity (as well as in transfection efficiency) was recently highlighted on a comparative study using these two cell lines, pointing out that a number of dissimilarities among these cell lines can account for this observation. Cellular death depends on factors such as how well individual cells are able to repair damage by active and passive mechanisms and the calcium concentration in the medium [62]. Studies to determine the ability of these materials to cross the cell membrane and release siRNA directly into the cytoplasm are needed to discern their mechanism of transfection.

## 4. Conclusions

We have evaluated the efficiency of two newly synthesized core-shell nanoparticles with a magnetic iron oxide core and a polycation surface coating (PEI-M/SiO_2_ and PHMBG-M/SiO_2_) as siRNA delivery vectors for magnetofection *in vitro*. In addition, this is the first report of PHMBG as siRNA carrier. Rational and successful design of optimized cationic polymer-based siRNA delivery vectors must consider two important factors: (i) enhanced transfection efficiency and (ii) toxicity reduction. Our study suggests that PEI-functionalized magnetic nanoparticles are promising candidates for nonviral siRNA delivery. They exhibit high transfection efficiency and are substantially less toxic than their nonmagnetic counterparts. The results here presented with PEI-M/SiO_2_ serve as model for the design of new materials and clearly demonstrate how magnetofection can be used to improve the material's transfection efficiency and since less dose is required the material's toxicity is also reduced.

## Figures and Tables

**Table 1:** Properties of the functionalized core-shell NP of the present study.

Particle species	Number-average hydrodynamic diameter (nm)^a^	Polymer content (wt%)^b^	Saturation magnetization (emu/g of magnetite)^c^
PHMBG-M/SiO_2_	160 ± 11	55–60	80–89
PEI-M/SiO_2_	240 ± 16	57–60	80–90

^a^Dynamic light scattering experiments were performed with a Brookhaven BI-200SM light scattering system (Brookhaven Instruments Corporation, Austin, TX) at a measurement angle of 90°. Particles dispersed in aqueous media (pH adjusted by 1 M NaOH or HCl) were filtered with a 0.45 *μ*m syringe filter prior to the DLS tests. The particles were dispersed with sonication in 10 mM KCl aqueous solution at approximately 0.05 wt% concentrations, and the pH of the nanoparticle suspensions was adjusted by adding 1 M HCl or NaOH aqueous solutions. Hydrodynamic diameter was measured in 10 mM KCl. ^b^The polymer and magnetite contents were found from elemental analyses and TGA. ^c^Saturation magnetization was found from SQUID measurements and divided per gram of iron (as found from elemental analysis) and then recalculated per gram of magnetite, assuming Fe_3_O_4_ structure of magnetite. No assumptions were required for the phase composition of the core material, as it had been previously proven to consist of magnetite.
